# Ketosis-Prone Diabetes in Severe Hypertriglyceridemia-Induced Pancreatitis: A Case Report

**DOI:** 10.7759/cureus.93035

**Published:** 2025-09-23

**Authors:** Abdullah M Almutairi, Sheima M Dahman, Mushabab A Alshahrani, Saleh N Alkurini

**Affiliations:** 1 Department of Internal Medicine, Division of Diabetes and Endocrinology, Security Forces Hospital, Riyadh, SAU; 2 Department of Family Medicine, King Fahad Medical City, Riyadh, SAU; 3 Department of Internal Medicine, Prince Faisal Bin Khalid Cardiac Center, Abha, SAU; 4 Department of Radiology, Security Forces Hospital, Riyadh, SAU

**Keywords:** diabetes, hypertriglyceridemia induced pancreatitis, ketosis prone diabetes, severe, severe diabetic ketoacidosis, types 2 diabetes

## Abstract

Diabetic ketoacidosis (DKA) is a serious acute complication commonly associated with type 1 diabetes mellitus (T1DM). It is characterized by elevated blood glucose levels, increased ketone production due to insufficient insulin, and metabolic acidosis. Acute pancreatitis (AP) is an inflammatory condition of the pancreas that can range from mild, self-limiting episodes to severe, life-threatening illness. The triad of DKA, hypertriglyceridemia, and AP presents a unique and challenging clinical scenario. This report describes the case of a 41-year-old female diagnosed with ketosis-prone diabetes and severe hypertriglyceridemia-induced pancreatitis. The case highlights several important considerations for clinicians and underscores the intricate interplay between metabolic disturbances, emphasizing the need for a comprehensive, multidisciplinary approach to management and treatment to address underlying causes and prevent future complications.

## Introduction

Type 2 diabetes mellitus (T2DM) is the most prevalent phenotypic form of diabetes in adults, distinguished by chronic hyperglycaemia arising from varying degrees of insulin resistance and insulin deficiency [1]. As per the report of the CDC (2024), in the United States, type 2 diabetes accounts for about 90% to 95% of all diagnosed cases of diabetes [2]. This condition, often linked to lifestyle factors such as obesity, sedentary behavior, and dietary habits, leads to a gradual decline in pancreatic beta-cell function, resulting in progressive insulin deficiency [3]. Unlike type 1 diabetes, which is an autoimmune disorder characterized by the destruction of pancreatic beta cells, T2DM involves a more complex interplay of genetic, environmental, and lifestyle factors contributing to its pathogenesis [4].

Diabetic ketoacidosis (DKA) is a serious acute complication commonly associated with type 1 diabetes mellitus (T1DM). It is characterized by elevated blood glucose levels, increased ketone production due to insufficient insulin, and metabolic acidosis [5]. However, it is becoming increasingly evident that DKA can also occur in individuals with T2DM, under certain conditions such as severe infections, myocardial infarction, or acute pancreatitis (AP), or in association with a distinct clinical entity known as ketosis-prone diabetes (KPD) [6,7].

KPD represents a unique and less familiar form of diabetes that bridges features of both T1DM and T2DM. Patients typically present with DKA, yet lack autoimmune markers of T1DM and may later achieve insulin independence, resembling T2DM in their long-term course [8]. KPD is classified according to the Aβ system into four subgroups based on the presence of islet autoantibodies (A⁺/A⁻) and β-cell functional reserve (β⁺/β⁻). Of these, the A⁻β⁺ subtype is the most common, presenting with DKA but often achieving insulin independence, whereas A⁻β⁻ patients remain insulin-dependent. This classification is clinically important as it helps predict prognosis, guide management decisions, and anticipate the likelihood of remission and the need for insulin therapy. KPD has been reported across diverse populations, accounting for up to 20-50% of DKA presentations in adults with type 2 phenotypes in multiethnic U.S. cohorts [9]. However, the concurrence of KPD with severe hypertriglyceridemia-induced pancreatitis is rarely reported, making this triad an unusual and clinically significant presentation.

AP is an inflammatory condition of the pancreas that can range from mild, self-limiting episodes to severe, life-threatening illnesses [10]. Hypertriglyceridemia, defined by abnormally high levels of triglycerides in the blood, is one of the major causes of AP, accounting for up to 10% of all cases. This condition can be exacerbated by poorly controlled diabetes, which often accompanies severe hypertriglyceridemia [11]. When present, hypertriglyceridemia can act as both a trigger and a consequence of metabolic dysregulation, amplifying systemic complications.

The risk of pancreatitis rises sharply when serum triglyceride levels exceed 1000 mg/dL, with the likelihood increasing further at concentrations above 2000 mg/dL [12]. The underlying pathophysiology involves several interrelated mechanisms. In severe hypertriglyceridemia, excessive chylomicron accumulation causes capillary hyperviscosity, leading to impaired pancreatic microcirculation and ischemia. Within the pancreas, triglycerides are hydrolyzed by pancreatic lipase into large amounts of free fatty acids, which, when unbound to albumin, exert direct cytotoxic effects on acinar cells and vascular endothelium. This cascade results in lipotoxic injury, further amplifying local inflammation, necrosis, and systemic complications. Understanding these mechanisms has direct therapeutic implications. Insulin not only lowers blood glucose but also activates lipoprotein lipase, accelerating the clearance of triglyceride-rich lipoproteins and reducing the pool of toxic free fatty acids. In refractory or severe cases with marked hypertriglyceridemia, systemic decompensation, or multiorgan involvement, plasmapheresis may be considered, as it rapidly removes chylomicrons and lowers plasma viscosity, thereby alleviating both ischemic and lipotoxic injury [13].

The triad of DKA, hypertriglyceridemia, and AP presents a unique and challenging clinical scenario. Each component of this triad can independently lead to significant morbidity and mortality, and their coexistence can complicate both diagnosis and management [14]. The occurrence of DKA in patients with T2DM and concurrent AP due to hypertriglyceridemia, although rare, underscores the necessity of a thorough understanding of the interplay between these conditions for effective clinical management [15].

This report presents the case of a 41-year-old female diagnosed with KPD with severe hypertriglyceridemia-induced pancreatitis. This case highlights several important considerations for clinicians. It emphasizes that DKA can occur in patients with type 2 diabetes, especially under severe physiological stress or in those with KPD. It demonstrates the significant, though uncommon, potential for AP to trigger DKA in the presence of hypertriglyceridemia. Lastly, the case underscores the need for a holistic treatment strategy that addresses all underlying conditions to achieve the best patient outcomes. This case adds to the literature on unusual diabetes presentations and emphasizes the importance of a comprehensive, multidisciplinary approach to managing such complex cases, ultimately improving patient care and outcomes.

## Case presentation

A 41-year-old Saudi married female with no prior medical history was admitted to the ED with a 24-hour history of abdominal pain that began abruptly while at rest. The pain was described as sharp (rated 7/10 in severity), stabbing, and initially localized to the left upper quadrant but radiated to the epigastric region and occasionally toward the back. She also experienced nausea but denied vomiting, diarrhea, constipation, urinary tract infection symptoms, or fever. There were no symptoms of hyperglycemia, such as polyuria, polydipsia, fatigue, or weight loss, nor any episodes of hypoglycemia. Her obstetric history included gestational diabetes during her first pregnancy in 2013, which was managed with insulin and resolved post-delivery. She had no gestational diabetes with her second child born in 2017. Her menstrual cycles were regular. At the time of presentation, she reported taking Metformin intermittently without physician supervision and GLP-1 receptor agonists (Tirzepatide and Semaglutide) for weight management three months earlier, which had been discontinued due to nausea and decreased appetite. She reported a dietary history of high-carbohydrate and fatty meals but denied alcohol intake or use of herbal supplements. The intermittent use of metformin and GLP-1 receptor agonists suggested underlying latent metabolic risk factors and highlighted the atypical nature of her presentation, given that she had not been formally diagnosed with diabetes nor maintained on regular therapy.

On examination, she was afebrile, with elevated blood pressure (156/89 mmHg) and tachycardia (heart rate of 132 bpm), but her oxygen saturation was normal. She appeared clinically dehydrated, with dry oral mucosa and reduced skin turgor. Abdominal examination revealed a soft abdomen with mild diffuse tenderness, most pronounced over the epigastrium and left lower quadrant, without guarding, rigidity, or rebound tenderness. Bowel sounds were present. No hepatosplenomegaly or palpable masses were noted. Initial laboratory investigations (Table 1) supported a diagnosis of moderate DKA. The rapid response team initiated the DKA protocol, and she was kept NPO with intravenous analgesia for pain management.

**Table 1:** Laboratory findings at admission (March 30, 2024) and at seven-week outpatient follow-up (May 19, 2024). GAD: Glutamic acid decarboxylase.

Lab Test	Initial Result (March 30, 2024)	Follow-Up (May 19, 2024)	Reference Range
Liver Function Tests			
Alanine aminotransferase (ALT)	4 U/L	15 U/L	7-56 U/L
Aspartate aminotransferase (AST)	7 U/L	17 U/L	10-40 U/L
Pancreatic Enzymes			
Lipase	185.20 U/L	53 U/L	13-60 U/L
Amylase	60 U/L	33 U/L	13-53 U/L
HbA1c	12.00%	7.50%	<5.7%
Venous Blood Gas (VBG)			
pH	7.18	7.44	7.35-7.45
Bicarbonate (HCO₃⁻)	11.7 mEq/L	33 mEq/L	22-28 mEq/L
Anion Gap	21 mEq/L	-	8-16 mEq/L
Blood Sugar Levels			
Blood Glucose	22.5 mmol/L	10.6 mmol/L	3.9-7.8 mmol/L (fasting)
Lipid Profile			
Triglycerides	44.58 mmol/L	1.64 mmol/L	<1.7 mmol/L
Cholesterol	16.54 mmol/L	4.54 mmol/L	<5.2 mmol/L
Low-density lipoprotein (LDL)	-	1.89 mmol/L	<3.5 mmol/L
High-density lipoprotein (HDL)	0.52 mmol/L	1.90 mmol/L	>1.68 mmol/L
C-Peptide	2.78 ng/mL	1.20 ng/mL	0.5-2.0 ng/mL
Urine Ketones	3	-	Negative
Antibodies			
Anti-GAD	0	-	Negative (<10.0)
Islet Cell Antibodies	0.00 nmol/L	-	≤0.02 nmol/L

**Figure 1:**
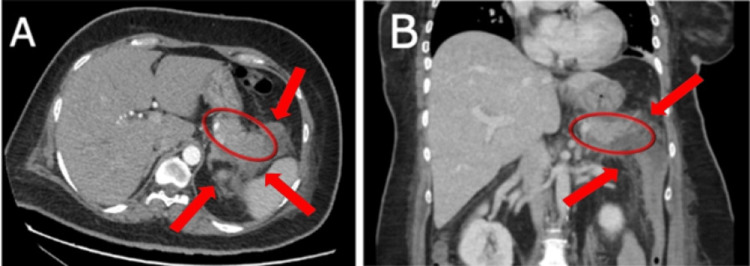
Contrast-enhanced computed tomography of the pancreas. A: Axial arterial-phase CT scan demonstrating a bulky pancreatic tail with ill-defined hypoenhancement on the arterial and portovenous phases (red oval), associated with peripancreatic fat stranding and free fluid (red arrows). B: Coronal portal venous-phase CT scan showing free fluid tracking along the left paracolic gutter (red arrows).

Further workup for abdominal pain revealed an elevated lipase level of 185.20 U/L (reference: 13.0-60.0 U/L), markedly elevated triglycerides at 44.58 mmol/L (reference <1.7 mmol/L), and cholesterol of 16.54 mmol/L (reference <5.2 mmol/L). Moreover, a contrast-enhanced CT scan of the pancreas (Figure 1) showed features of pancreatic tail focal interstitial pancreatitis without complications. Based on these findings, the patient was diagnosed with ketosis-prone diabetes and pancreatitis secondary to hypertriglyceridemia. These findings prompted urgent ICU admission for closer monitoring and care. Under the endocrine team's management, she was transitioned to a basal/bolus insulin regimen. Gastroenterology advised keeping her NPO, ensuring good hydration, and monitoring her oral intake tolerance. DKA resolved, coinciding with normalization of blood pH and ketone clearance, along with a downward trend in triglycerides (Table 1). Consequently, the patient was transferred from the ICU to the medical ward. Lipase levels also began to decline, paralleling clinical improvement in abdominal pain. Initial C-peptide levels were 2.78 ng/mL, decreasing to 1.13 ng/mL, suggesting reduced endogenous insulin production. Glutamic acid decarboxylase-65 and islet cell antibodies were negative, indicating no autoimmune beta-cell destruction. Despite resolving pancreatitis, the patient had persistent hyperglycemia requiring daily insulin degludec and pre-meal insulin aspart.

After stabilizing, she was discharged with an insulin multiple daily injection regimen, based on persistently reduced C-peptide (1.20 ng/mL) and negative islet autoantibodies, and was scheduled for follow-up with a diabetologist, diabetic educator, and clinical nutritionist. One month later, her blood glucose levels ranged between 100 and 180 mg/dL on insulin, and her triglycerides had decreased by more than 96% (from 44.58 to 1.64 mmol/L), well beyond laboratory variability and indicating a clinically meaningful improvement. Total cholesterol declined by approximately 72% (from 16.54 to 4.54 mmol/L), and LDL fell to 1.90 mmol/L, within the target range (<3.5 mmol/L). Similarly, her HbA1c improved from 12.0% to 7.5% (a 37.5% relative reduction, corresponding to a 4.5 percentage point absolute decline), consistent with substantial improvement in glycemic control. Following triglyceride reduction to near-normal levels (1.64 mmol/L) and cholesterol decline to 4.54 mmol/L with LDL 1.90 mmol/L, atorvastatin was initiated to maintain long-term lipid control. Furthermore, the improvement in HbA1c from 12.0% to 7.5% over four weeks supported the addition of metformin to reduce insulin requirements and enhance glycemic durability.

This case underscores the complexities of diagnosing and managing DKA in the presence of acute pancreatitis, especially when secondary to hypertriglyceridemia. Despite the challenges, comprehensive care and close follow-up ensured her condition stabilized, highlighting the importance of multidisciplinary management in such cases. Table 1 summarizes the critical lab findings during patient admission, treatment, and follow-up, illustrating the complex interplay of DKA, hypertriglyceridemia, and pancreatitis, as well as the effectiveness of the management strategies employed. All laboratory results presented in Table 1 were obtained under fasting conditions unless otherwise specified. No values were indeterminate or outside assay detection limits, and repeat triglyceride testing on admission confirmed the extreme elevation, excluding postprandial variability.

## Discussion

This case highlights the complex interplay between DKA, hypertriglyceridemia, and acute pancreatitis (AP) in a patient with ketosis-prone diabetes (KPD). KPD, unlike classical T1DM, can present with severe insulin deficiency that may remit after initial insulin therapy. This case highlights several critical learning points. Firstly, KPD should be considered in patients presenting with DKA who do not display the classical features of type 1 diabetes, particularly those with a history of gestational diabetes or inconsistent diabetes management, as noted in various studies [16]. Secondly, the prompt recognition and management of hypertriglyceridemia are essential to prevent the onset of AP and DKA. Effective therapeutic strategies may include the use of lipid-lowering agents, dietary modifications, and, in severe cases, plasmapheresis. Additionally, managing complex metabolic conditions, such as the combination of DKA, hypertriglyceridemia, and AP, requires a multidisciplinary approach. Collaboration across multiple specialties is crucial to ensure comprehensive care and achieve optimal patient outcomes. Finally, it is important to regularly monitor blood glucose, HbA1c, triglyceride levels, and other key indicators to assess treatment effectiveness and adjust management plans accordingly.

KPD is particularly prevalent in individuals of African, Hispanic, or Asian descent [17,18]. The decision to discontinue insulin therapy in patients with KPD should be guided by a precise classification of the KPD subtype and an evaluation of predictive factors, ideally conducted during the initial outpatient visit, 1-3 weeks post-hospital discharge. To date, four primary classification schemes have been developed to stratify KPD patients into distinct clinical subgroups: the American Diabetes Association (ADA) system, a modified ADA system, a BMI-based system, and the Aß system, which categorizes patients based on the presence or absence of autoantibodies and the beta-cell functional reserve. In a longitudinal study comparing the four classification schemes for accuracy and predictive value, the Aß system was shown to be the most accurate in predicting long-term insulin dependence 12 months after the index DKA event, with 99% sensitivity and 96% specificity [19,20]. This classification system divides patients into four subgroups: A+ß- (autoantibodies present, beta-cell function absent), A+ß+ (autoantibodies present, beta-cell function present), A-ß- (autoantibodies absent, beta-cell function absent), and A-ß+ (autoantibodies absent, beta-cell function present).

According to the Aß system classification, as shown in Table 2 [21], the patient aligns with the A-ß+ subgroup, which constitutes the largest proportion of KPD patients (approximately 50%) in multiethnic cohorts in the United States. They present with DKA yet have the clinical features and subsequent behavior of type 2 diabetes [22]. The management of KPD, AP, and hypertriglyceridemia involves a multifaceted approach. For KPD, treatment typically starts with acute management of DKA, insulin therapy to control blood glucose levels, and may include medications to manage ketosis [23]. Outpatient management should begin shortly after resolution of DKA, including classification of the patient according to KPD subgroup and evaluation of predictive factors such as beta-cell function, beta-cell autoimmunity, and, in some instances, HLA typing. For long-term management, patients should receive regular counseling from a nutritionist and diabetic educator. It is recommended that they engage in at least 150 minutes of physical activity per week, and weight loss may be encouraged for obese ß+ individuals. Smoking cessation should be reinforced. Additionally, patients should undergo routine screening and receive appropriate treatment for microvascular and macrovascular complications of diabetes, following the guidelines set by the ADA [24].

**Table 2:** Aß classification of ketosis-prone diabetes (KPD). Source: Reference [21]. DKA: Diabetic ketoacidosis; T2DM: Type 2 diabetes mellitus.

Subgroup	Autoantibodies (A)	β-cell Function (ß)	Clinical Features	Clinical Implications
A+ß-	Present	Absent	Classic autoimmune type 1 diabetes	Lifelong insulin dependence
A+ß+	Present	Preserved	Autoimmune diabetes with residual function	May have partial remission; often progress to insulin dependence
A-ß-	Absent	Absent	Idiopathic insulin-deficient diabetes	Require long-term insulin therapy
A-ß+	Absent	Preserved	“True” ketosis-prone type 2 diabetes; most common (~50%)	Present with DKA but may discontinue insulin and be managed like T2DM

AP management focuses on supportive care, including fluid resuscitation, pain control, and nutritional support, while addressing the underlying cause, such as hypertriglyceridemia [25]. Hypertriglyceridemia is managed through lifestyle modifications, including diet and exercise, and pharmacotherapy such as fibrates or statins, if necessary [16]. Effective therapeutic strategies may involve the use of lipid-lowering medications and dietary changes. Plasmapheresis is typically considered when triglyceride levels are above 1000 mg/dL in the setting of pancreatitis with worsening systemic inflammatory response syndrome (SIRS) (Table 3) [26], organ dysfunction and/or multi-organ failure (Table 4) [27], or if triglycerides fail to respond to standard therapy [28]. If plasmapheresis is not available or is poorly tolerated, insulin may be utilized. Insulin promotes the activation of lipoprotein lipase (LPL) in muscle and adipose tissues, facilitating the breakdown of chylomicrons [29].

**Table 3:** Signs of systemic inflammatory response syndrome (SIRS). Reference: [26]. Reproduced with permission from BMJ Publishing Group Ltd. Originally published in [26]. License number: 6058941040097, June 30, 2025.

Criteria	Description
Heart rate	>90 beats/min
Core temperature	<36 °C or >38 °C
White blood cell count	<4,000/mm³ or >12,000/mm³
Respiratory rate	>20 breaths/min or PaCO₂ <32 mmHg
SIRS: defined by the presence of two or more criteria	

**Table 4:** Modified Marshall scoring system for organ dysfunction. A score of ≥2 in any system defines the presence of organ failure. Organ failure resolving within ≤48 hours is defined as transient organ failure; organ failure persisting for >48 hours is defined as persistent organ failure. *Without inotropic agents.

Organ system	Score: 0	1	2	3	4
Respiratory (PaO₂/FiO₂)	>400	301-400	201-300	101-200	≤100
Renal (serum creatinine, mg/dL)	<1.4	1.4-1.8	1.9-3.6	3.6-4.9	>4.9
Cardiovascular (systolic blood pressure, mmHg)*	>90	<90, fluid responsive	<90, not fluid responsive	<90, pH <7.3	<90, pH <7.2

Management of hypertriglyceridemia-induced AP entails treating the AP itself and implementing strict dietary fat restriction, with the objective of lowering serum triglyceride levels to below 500 mg/dL (5.6 mmol/L) [30]. Once triglyceride levels fall below this threshold, patients will require long-term therapy to prevent recurrent pancreatitis and to mitigate other complications associated with hypertriglyceridemia. This consists of both lifestyle modifications, including diet and exercise, and pharmacotherapy such as fibrates or statins, if necessary.

Addressing all three conditions simultaneously requires careful coordination to prevent complications and improve patient outcomes. The successful management of this case underscores the importance of early recognition and treatment of hypertriglyceridemia-induced AP in patients with KPD. This rare but significant clinical scenario demands a thorough understanding of the interplay between metabolic and inflammatory processes and highlights the value of a multidisciplinary approach in achieving favorable patient outcomes.

## Conclusions

The patient’s diagnosis reveals a complex metabolic condition characterized by KPD, in which the individual experiences episodes of severe hyperglycemia and ketosis similar to type 1 diabetes but without typical autoimmune markers. In addition, the patient developed pancreatitis, a serious inflammation of the pancreas triggered by hypertriglyceridemia, a condition marked by extremely high levels of triglycerides in the blood. This combination highlights the intricate interplay of metabolic disturbances and emphasizes the need for a comprehensive, multidisciplinary approach to management. The case also underscores the importance of early screening and follow-up in women with a history of gestational diabetes to reduce the risk of such challenging presentations. As this report reflects a single case, its findings cannot be generalized to all patients. Longer-term monitoring of β-cell function and lipid control will be essential to determine the sustainability of metabolic recovery and to guide ongoing care.
